# Kidney Biopsy in a Pregnant Patient with Suspected Glomerular Disease: CON

**DOI:** 10.34067/KID.0000000000000243

**Published:** 2023-10-26

**Authors:** Emily M. Moss, Ursula C. Brewster

**Affiliations:** Department of Internal Medicine, Yale University School of Medicine, New Haven, Connecticut

**Keywords:** clinical nephrology, glomerular disease, kidney biopsy

Kidney biopsy is considered the gold standard for diagnosis of suspected glomerulopathies, although its use in pregnancy remains controversial. Kidney biopsy may be indicated in pregnant patients for evaluation of sudden and severe drop in kidney function, new development of nephrotic range proteinuria, or to diagnose a suspected *de novo* or recurrent lupus nephritis flare for which histopathologic information is required to guide appropriate management. However, despite its potential utility for assessment of glomerular disease, kidney biopsy is associated with increased morbidity in both mother and fetus after the first trimester and should be avoided in most circumstances.

**Figure 1:**
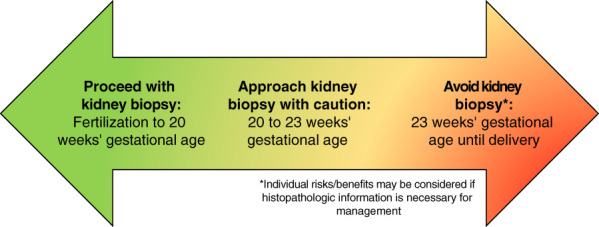
**Simplified approach to pursuing kidney biopsy in pregnancy according to gestational age.** *Individual risks/benefits may be considered if histopathologic information is necessary for management.

While current data are largely limited to single-center experiences, studies show that kidney biopsy in pregnancy is generally safe until a gestational age of 20 weeks. A systematic review demonstrated that major complications from biopsy (*e.g.*, retroperitoneal or perirenal hematoma requiring transfusion, placental abruption, or preterm delivery) occurred after this time at a median of 25 weeks of gestation (range 23–26 weeks).^1^ Recent clinical practice guidelines from the UK Renal Association support the pursuit of kidney biopsy in the first and early second trimester of pregnancy but only if a histological diagnosis will change management.^2^ A simplified approach to kidney biopsy in pregnancy is summarized in Figure 1.

There are several key reasons why kidney biopsy should be approached with extreme caution in pregnancy. First, biopsy in these patients carries increased procedural risks because of the anatomic and physiologic changes that occur in pregnancy. In normal pregnancy, widespread hemodynamic changes such as vasodilation, volume expansion, and increased cardiac output lead to significant increases in renal plasma flow and eGFR.^3^ As a result of increased renal plasma flow, pregnant patients are at greater risk of major (*e.g.*, perinephric or retroperitoneal hematoma requiring blood transfusion) and minor (*e.g.*, microhematuria or macrohematuria) complications.^1^ This can be further exacerbated in cases of difficult-to-control hypertension, a feature often seen in pregnant adults for whom kidney biopsy is indicated.

Second, positioning of the pregnant patient can increase technical difficulty of the procedure. As the gravid uterus grows in size, standard prone positioning is usually not tolerated by patients past 20–25 weeks of gestation. Clinicians have less procedural experience with alternative positions such as lateral decubitus or seated positions which may lead to higher complication rates.^4,5^ Moreover, one systematic review found that despite incorporation of ultrasound guidance into practice, there seems to be no reduction in the risk of kidney biopsy–related complications in pregnant patients from study periods of 1966–1981 to 2007–2008.^1^

Perhaps the most somber risk of kidney biopsy in pregnancy is a fetal adverse event. Cases of placental abruption, preterm delivery, and presumed fetal death have resulted from biopsy in the third trimester, although the relationship between these obstetric complications and biopsy itself (compared with underlying disease process) has been questioned.^1^ Reports of such adverse events have led some researchers to deem kidney biopsy in pregnancy a morbid procedure.^6^ One study reviewed outcomes of 20 women who underwent antepartum biopsy and 75 women who underwent kidney biopsy in the *postpartum* period. The results showed that women with more severe kidney disease (as defined by Modification of Diet in Renal Disease estimation of GFR) at the time of biopsy had increased risk of obstetrical complications (*e.g.*, preeclampsia, preterm delivery, or delivery through caesarean section), suggesting an association between disease morbidity and risk of biopsy.^7^ To this end, the perceived benefit of kidney biopsy for diagnostic purposes must be weighed heavily against potential risks to mother and fetus. A brief summary of the risks of kidney biopsy in pregnancy is highlighted in Table 1.

**Table 1:** Summarized risks of kidney biopsy in late pregnancy

Risks	Etiology
Bleeding complications	• Increased renal plasma flow contributes to risk of perinephric or retroperitoneal hematoma requiring blood transfusions
Increased technical difficulty of procedure	• Typical prone positioning is often not tolerated by pregnant patients past early second trimester requiring alternative positioning • Clinicians have less experience with alternative positioning which may lead to higher complication rates • Introduction of ultrasound has not reduced complications in pregnancy
Additional harm to fetus	• Case reports of placental abruption, preterm delivery, and presumed fetal death
Alternative options to kidney biopsy	• Biopsy before 20 wk of gestational age • Consider utility of noninvasive serum diagnostic markers • Delay biopsy to *postpartum* period

In most cases of suspected glomerulopathies, seeking a definitive diagnosis through kidney biopsy after the early second trimester should be deferred to the *postpartum* period. Renal function can be closely monitored by serum and urine testing during the last trimester until fetal maturity and delivery. Benefits of postponing biopsy were clear in one systematic review, featuring 243 biopsies performed in pregnancy and 1236 biopsies performed after delivery. Data revealed that patients who underwent antepartum biopsy had an increased risk of all major and minor complications compared with *postpartum* biopsy (7% versus 1%, *P* = 0.001).^1^ An obvious exception to delaying biopsy is in cases of suspected lupus nephritis with high disease activity when histologic information is needed to determine proper therapy. But much of the time, when that is the case later in pregnancy, delivery (naturally or by induction) is imminent, so one should consider empiric steroid therapy for a short period of time until the baby is safely born and then biopsy *postpartum*.

Advances in noninvasive diagnostic testing may also quell the necessity of antepartum kidney biopsy. Utility of serum markers for diagnosis of anti–M-type phospholipase A2 receptor in membranous nephropathy and anti-suPAR (soluble urokinase plasminogen activator receptor) in focal segmental glomerular sclerosis have been reported, although their use in pregnancy requires further study.^8,9^ Recent investigations have also detailed novel serum or urine biomarkers for conditions such as preeclampsia and IgA nephropathy, but data are still limited. Historic serologies such as those for vasculitis (*e.g.* antineutrophil cytoplasmic antibody) and lupus (*e.g.* antinuclear antibody, anti–double stranded DNA antibody) may be useful to guide diagnosis without tissue sampling with the caveat of differential management in subclasses of lupus nephritis mentioned above. Because pregnancy can be considered an acute stress on the body, falling compliment levels, which are typically normal or high in pregnancy, can signal heightened lupus disease activity.

In sum, kidney biopsy for the evaluation of suspected glomerular disease is generally safe in pregnancy until 20 weeks of gestation. After this time, risks of biopsy increase because of technical challenges with patient positioning and physiologic changes specific to pregnancy. The most commonly reported adverse events involve bleeding (*e.g.*, hematoma or hematuria) but can be as serious as placental abruption and preterm delivery. Severity of underlying kidney disease may also increase risk of kidney biopsy. To avoid these morbid complications, kidney biopsy in the late second or third trimester should be delayed until after delivery whenever possible.
